# RELIGION, SOCIOECONOMIC HETEROGENEITY, AND COGNITIVE STATUS AMONG BLACK MEN

**DOI:** 10.1093/geroni/igae098.1596

**Published:** 2024-12-31

**Authors:** Marino Bruce, Bettina Beech, Dylan Tran, Baasil Kaka, Roland Thorpe, Jr.

**Affiliations:** University of Houston, Houston, Texas, United States; University of Houston, Houston, Texas, United States; University of Houston, Houston, Texas, United States; University of Houston, Houston, Texas, United States; Johns Hopkins University, Baltimore, Maryland, United States

## Abstract

Risks for cognitive impairment are disproportionately high among Black men, however religious practices and beliefs have been shown to be protective for members of this group with modest incomes, suggesting that social class can complicate this association. The purpose of this study was to examine the association between religious practices and/or beliefs, socioeconomic status (SES), and cognitive status among Black men in the 2016 Health and Retirement Study (HRS). Data were drawn from 384 Black men who completed the Core and Leave Behind Questionnaires. The primary outcome was any cognitive impairment, a dichotomous variable derived from a modified version of the Telephone Interview for Cognitive Status (TICS). Religious practices and beliefs (private prayer, religious beliefs) and SES (income, education) were primary independent variables. Descriptive results indicate that individuals reporting incomes less than $50,000 had a higher proportion of any cognitive impairment than respondents reporting higher incomes (48.2% vs 19.1%). Differences in any cognitive impairment were also found between respondents reporting 12 or fewer years of education and individuals reporting more than twelve years of education (48.8% vs 19.8%). Data from modified Poisson regression models indicated that personal private prayer (PR=0.67, CI:0.47-0.97) and religious beliefs (PR=0.92, CI:0.84-1.00) were beneficial for Black men with modest incomes. Private prayer was inversely related to any cognitive impairment among Black men with more than 12 years of education (PR=0.51, CI:0.27-0.99). Full-scale studies of Black men are needed to further examine how SES can influence factors that may be protective against cognitive impairment among this population.

